# FAK PROTAC Inhibits Ovarian Tumor Growth and Metastasis by Disrupting Kinase Dependent and Independent Pathways

**DOI:** 10.3389/fonc.2022.851065

**Published:** 2022-04-28

**Authors:** Xueyun Huo, Wenjing Zhang, Guannan Zhao, Zhenwen Chen, Peixin Dong, Hidemichi Watari, Ramesh Narayanan, Todd D. Tillmanns, Lawrence M. Pfeffer, Junming Yue

**Affiliations:** ^1^ School of Basic Medical Sciences, Capital Medical University, Beijing, China; ^2^ Department of Pathology and Laboratory Medicine, College of Medicine, University of Tennessee Health Science Center, Memphis, TN, United States; ^3^ Center for Cancer Research, College of Medicine, University of Tennessee Health Science Center, Memphis, TN, United States; ^4^ Department of Genetics, Genomics & Informatics, College of Medicine, University of Tennessee Health Science Center, Memphis, TN, United States; ^5^ Department of Gynecology, Hokkaido University School of Medicine, Hokkaido University, Sapporo, Japan; ^6^ Department of Medicine, College of Medicine, University of Tennessee Health Science Center, Memphis, TN, United States; ^7^ Division of Gynecologic Oncology, Department of Obstetrics and Gynecology, University of Tennessee Health Science Center, West Cancer Center, Germantown, TN, United States

**Keywords:** ovarian cancer, focal adhesion kinase (FAK), FAK PROTAC, ASAP1, metastasis

## Abstract

Focal adhesion kinase (FAK) is highly expressed in a variety of human cancers and is a target for cancer therapy. Since FAK kinase inhibitors only block the kinase activity of FAK, they are not highly effective in clinical trials. FAK also functions as a scaffold protein in a kinase-independent pathway. To effectively target FAK, it is required to block both FAK kinase-dependent and FAK-independent pathways. Thus, we tested a new generation drug FAK PROTAC for ovarian cancer therapy, which blocks both kinase and scaffold activity. We tested the efficacy of FAK PROTAC and its parent kinase inhibitor (VS-6063) in ovarian cancer cell lines *in vitro* by performing cell functional assays including cell proliferation, migration, invasion. We also tested *in vivo* activity in orthotopic ovarian cancer mouse models. In addition, we assessed whether FAK PROTAC disrupts kinase-dependent and kinase-independent pathways. We demonstrated that FAK PROTAC is highly effective as compared to its parent FAK kinase inhibitor VS-6063 in inhibiting cell proliferation, survival, migration, and invasion. FAK PROTAC not only inhibits the FAK kinase activity but also FAK scaffold function by disrupting the interaction between FAK and its interaction protein ASAP1. We further showed that FAK PROTAC effectively inhibits ovarian tumor growth and metastasis. Taken together, FAK PROTAC inhibits both FAK kinase activity and its scaffold protein activity by disrupting the interaction between FAK and ASAP1 and is highly effective in inhibiting ovarian tumor growth and metastasis.

## Introduction

Ovarian cancer (OC) is the deadliest women’s malignancy with a five-year survival rate of less than 39 and 17% at stage III and IV, respectively (1). It is challenging to treat OC because of aggressive peritoneal metastasis and chemoresistance. FAK (Focal adhesion kinase), encoded by the *PTK2* (protein tyrosine kinase) gene is highly expressed in multiple cancers including OC, and is associated with tumor metastasis and chemoresistance (2–6). Targeting FAK for cancer therapy has been extensively investigated and FAK kinase inhibitors including VS6063, GSK2256098 were tested in phase I or phase II clinical trials. However, FAK kinase inhibitors only showed modest effects and have not received FDA approval for cancer therapy (7–9). FAK functions through kinase-dependent and kinase-independent pathways (10, 11). FAK inhibitors only inhibit FAK kinase activity (FAK kinase-dependent pathway). However, they do not block the kinase-independent pathway, in which FAK functions as a scaffold protein and participates in protein-protein interactions (12). Therefore, an effective drug to target FAK needs to block both kinase-dependent and kinase-independent pathways.

Recently, FAK PROTAC (Proteolysis Targeting Chimeric Molecule) degrader has been developed to block both pathways and showed greatly improved activity to inhibit cancer cell invasion as compared to its parent VS6063 kinase inhibitor (12). PROTACs are designed to degrade target proteins through the ubiquitin-proteasome pathway. The PROTAC molecule covalently binds to protein of interest (POI) on one end and binds to an E3 ligase on the other end, and then transfer ubiquitin from an E2 enzyme to the POI, thus results in the POI’s polyubiquitination, and subsequently degradation through the proteasome recognition (12–14). PROTACs are highly effective in chemically knocking down its target protein (15–19). PROTAC based drugs have been in clinical trials and showed great efficacy in cancer therapy (15, 20). FAK PROTAC, which was designed based on VS6063 inhibitor that was already in clinical trials, is highly effective in degrading FAK protein in breast cancer cells (12).

FAK has multiple function domains, including a protein 4.1-ezrin-radixin-moesin (FERM) domain, a kinase domain, a focal adhesion targeting (FAT) domain, and three proline-rich (SH) regions. FAK is activated by autophosphorylation at Y397 position and controls cellular events including cell proliferation, migration, and survival, through the kinase-dependent pathway. FAK also functions through a kinase-independent pathway, which is mediated by FERM, FAT, or SH domains (21). FAK contains nuclear export signals (NES) and nuclear localization signals (NLS) (21), which allows FAK to transport in and out of the nucleus and participate in gene and protein regulation. Previous studies showed that FAK directly interacts with p53 or ASAP1 (ADP ribosylation factor ARF GTPase-activating protein GAP containing SH3, ANK repeats, and PH domain), which does not require FAK kinase activity (22). FAK interacts with ASAP1 through its SH3 domain with the pleckstrin homology (PH) of ASAP1 (23, 24). ASAP1 is aberrantly expressed in different cancer types including OC (25–28). Interestingly, both *PTK2* and *ASAP1* are located in an oncogenic genomic locus 8q24 and are associated with tumor metastasis and recurrence (27, 28). We recently reported that FAK is co-expressed and interacts with ASAP1 in OC (3), which may regulate ovarian tumor metastasis and chemoresistance.

In this study, we investigated the role of FAK in kinase-dependent and kinase-independent pathways by focusing on FAK kinase activity and the interaction between FAK and ASAP1 and tested the efficacy of FAK PROTAC in OC cell lines and orthotopic OC mouse model. Our findings demonstrated that FAK PROTAC is highly effective in inhibiting OC cell invasion and metastasis by blocking FAK kinase activity and disrupting the interaction between FAK and ASAP1.

## Materials and Methods

### Cell Culture

OC OVCAR3 and OVCAR8 cell lines were purchased from the National Cancer Institute and maintained in RPMI 1640 medium supplemented with 10% FBS (Atlanta Biologicals, GA), 100 U/ml penicillin, and 100 μg/ml streptomycin (Fisher Scientific) in a humidified incubator at 37°C in 5% CO_2_. VS6063 and FAK PROTAC Degrader 1 was purchased from MedChemExpress (MCE).

### Cell Proliferation Assay and Cell Viability

Cell proliferation was examined following instructions of MTT assay kit (ATCC, Manassas, VA). Briefly, OVCAR3 or OVCAR8 cells (3,000/well) were plated into 96-well plates and treated with different doses of FAK PROTAC, VS6063 or vehicle for various time points, and then MTT reagent (5mg/mL) was added to each well. After a 4-hour incubation, the media/MTT was aspirated and 100 μL DMSO was added to each well. The plate was incubated for 20 min and the absorbance was measured at single-wavelength mode (490 nm) using a Multiskan MK3 Microplate Reader (Thermo Fisher Scientific). BrdU proliferation assay was performed by labelling OC cells for 12 h with 10 µM BrdU and cells were fixed with 3.7% formalin and cells were washed and incubated with BrdU antibody (Santa Cruz, Cat.No. SC-32323) for 24h at 4°C and then fluorescent labelled secondary antibody Alexa 488 (Thermo Fisher Scientific. Cat. No. A32723) for 1h at room temperature. Cell proliferation was detected by counting BrdU labelled cells and DAPI labelled cell nuclei from four different fields under fluorescent microscopy.

### Cell Clonogenic Assay

OVCAR3 or OVCAR8 cells (300 cells/well) were plated into 6-well plates and treated with 0.5 μM FAK PROTAC, VS6063 or vehicle for two weeks and then stained with 0.1% Crystal Violet. Cell colonies were counted using ImageJ software from three different wells.

### Cell Migration Assay

Cell migration was performed using modified Transwell chambers (BD Falcon™, San Jose, CA) inserted into 24-well culture plates as described previously (3). Briefly, OVCAR3 or OVCAR8 cells were treated with 1 μM FAK PROTAC, VS6063 or vehicle. After 24h cells were seeded into transwells. RPMI 1640 containing 10% FBS was added in the bottom of the chamber as the chemical attractant and incubated for 16 h. The upper chamber medium and non-migrated cells were removed, and migrated cells were fixed with methanol and stained with crystal violet. Images were taken at 10× magnification and migrated cells were counted in at least three randomly selected different fields using the ImageJ software. Cell migration was also determined by wound healing assays as described previously (29). Briefly, cells (3×10^5^ cells per well) were seeded in triplicate into 6-well plates and cultured for 24h, and then cell surface was scratched with a pipette tip. After 24h of culture, the migration rate was calculated using the following formula: (area of the wound area at 0 h - the wound area at 24 h)/the wound area at 0 h.

### Cell Invasion Assay

OVCAR3 or OVCAR8 cells (3×10^5^) were treated with 1 μM FAK PROTAC, VS6063 or vehicle for 24 h and then seeded in serum-free RPMI 1640 media onto Matrigel (BD BioSciences, San Jose, CA) coated transwells. RPMI 1640 containing 10% FBS was added in the bottom chamber as chemoattractant and incubated for 24h. The invaded cells were stained with H&E and counted as described previously (30).

### Immunofluorescent Staining

Paraffin-embedded ovarian sections of HGSC were obtained from de-identified ovarian patient biopsy specimens (UTHSC Tissue Services Core). Deidentified ovarian tumor PDX sections and human OC specimens were collected under a UTHSC IRB-approved protocol (14-03113-XP). Sections were subjected to antigen retrieval at 90°C for 30 mins, and then incubated with FAK (Cell Signaling Technology, Inc, Danvers, MA) and PCNA (Santa Cruz, CA, Cat.No. SC-56) antibodies at 4°C overnight. Sections were washed and incubated with secondary antibody Alexa 488 or 594 conjugated goat anti-rabbit (1:200 dilution, Fisher Scientific). Nuclei were counterstained with DAPI.

### Immunohistochemical Staining

Paraffin-embedded ovarian tumor sections were subjected to antigen retrieval at 90°C for 30 mins and incubated at 4°C overnight with FAK (Cell Signaling Technology, Inc, Danvers, MA) and PCNA (Santa Cruz, CA) antibodies, and then incubated with biotinylated secondary antibody using the Vectastain Elite kit (Vector Laboratories).

### Western Blot (WB)

WB was performed as described previously (3). The membranes were blocked with 5% nonfat milk for 1h and incubated with primary antibodies against ASAP1 (1:1,000, Santa Cruz, CA, Cat.No.SC32323), FAK (Cat.No. 3285S), p-FAK, (1:1000, Cell Signaling Technology, Inc, Danvers, MA, Cat. No.8556S) and GAPDH (1:1,000, Sigma, St. Louis, MO, Cat. No. G8795) overnight at 4°C. The membranes were washed three times with PBST and then incubated with HRP-conjugated secondary antibodies (Yeasen) for 1 h. After the final wash with TBST, the proteins were detected using enhanced chemiluminescence (ECL) reagents.

### Immunoprecipitation (IP)

Cells were treated with 1 µM FAK PROTAC, VS6063 and vehicle for 24 h and then cells were collected for IP as described previously (3) using the Pierce Classic IP Kit (Thermo Fisher). Cell lysate was incubated with either 10 µg of IgG or ASAP1 antibody (Santa Cruz, Cat. NO. SC-374410) overnight at 4°C. The immune complex was then captured with Pierce Protein A/G Agarose beads for 1h at 4°C, then washed with lysis buffer and conditioning buffer, and eluted with elution buffer. The immune complex was analyzed by WB using ASAP1 or FAK antibodies.

### Mouse Xenograft Model

All animal experiments were conducted in accordance with the approval from the Institutional Animal Care and Use Committee (IACUC) at the University of Tennessee Health Science Center. Luciferase reporter gene labelled OVCAR8 cells were intrabursally injected into ten two-month-old immunocompromised NOD scid gamma (NSG) female mice and randomly divided into two groups. Mice were treated with FAK PROTAC (10 mg/kg body weight) or vehicle every day for three weeks. Tumor growth and metastasis were monitored using a Xenogen live animal imaging system weekly. All mice were sacrificed after three-weeks of treatment, tumors were weighed and collected for WB to detect FAK.

### Database Query

To examine the genetic alterations and expression of *PTK2* in OC, we queried the TCGA (https://tcga-data.nci.nih.gov/tcga/tcgaHome2.jsp) and Oncomine database (https://www.oncomine.org). For gene copy number analysis, OC patient data were selected from Pan Cancer Atlas and compared to other cancer types in TCGA database. In Oncomine database, OC patient samples were selected from two TCGA cohorts including normal ovaries, normal blood and HGSC. Copy number or expression level was analyzed by selecting bar-graph format.

### Statistical Analysis

Significant differences between independent experiments were determined by one-way ANOVA or Student’s *t*-test, and the data were expressed as mean ± SD. *P*<0.05 was considered as statistically significant.

## Results

### *PTK2* Expression Is the Highest in OC and Is Associated With Patient Poor Survival

**Figure 1:**
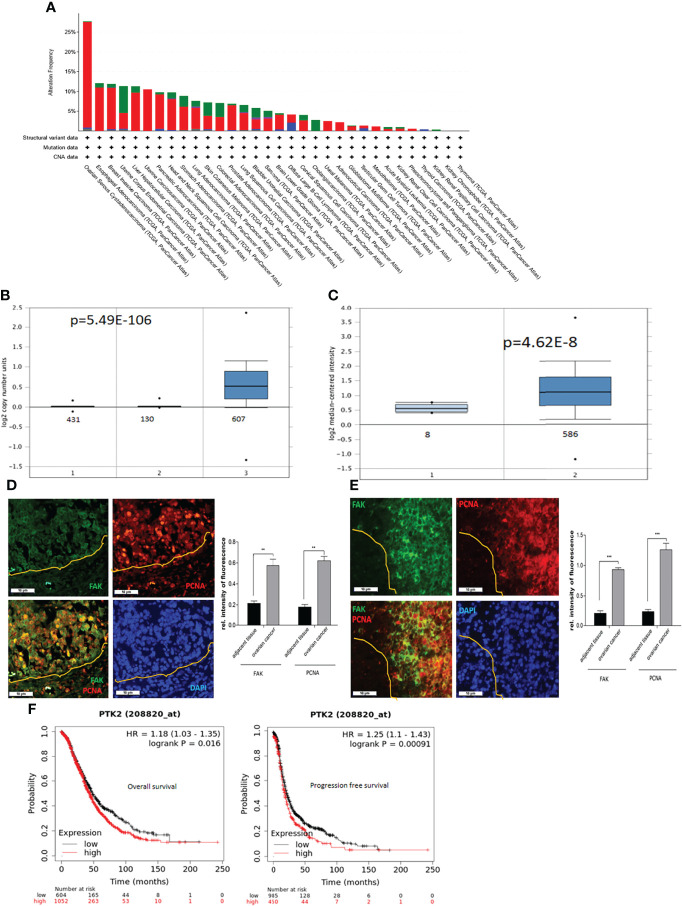
*PTK2* is highly expressed in OC and correlated with OC patient poor survival. **(A)** *PTK2* alteration frequencies in OC compared to other cancer types in TCGA. **(B)** *PTK2* copy numbers were shown in 607 OC compared to 431 normal blood and 130 ovaries in Oncomine. **(C)** *PTK2* expression is significantly upregulated in 586 OC compared to that in 8 normal ovaries in Oncomine. **(D, E)** Immunofluorescent staining of FAK (green) and PCNA (red) in HGSC **(D)** or PDX **(E)** sections. Nuclei were counterstained with DAPI (blue). Fluorescent intensity was quantified by Image J. **(F)** Survival analysis of OC patients from Kaplan-Meier database. Both overall and PFS survival rates were analyzed from 1656 OC patents (*p<0.05; **p<0.01; ***p<0.001).

To assess the expression of *PTK2* in cancer, we examined gene profiles from dataset in the TCGA’s Pan Cancer Atlas (The Cancer Genome Atlas). *PTK2* is most upregulated or amplified in OC, followed by esophageal and breast cancer among 32 cancer types listed in **Figure 1A**. We then examined the copy numbers of *PTK2* in 431 samples of normal blood, 130 samples of normal ovaries and 607 samples of high-grade serous carcinoma (HGSC) and found *PTK2* was significantly amplified or upregulated in HGSC compared to normal blood or ovarian samples (**Figure 1B**). We then examined *PTK2* expression is 586 samples of HGSC and 8 samples of normal ovaries in the Human Genome U133A Array data (https://www.oncomine.org). *PTK2* is significantly higher in HGSC than that in normal ovaries (**Figure 1C**). To validate PTK2 (FAK) expression, we perform immunostaining on sections of human HGSC (**Figure 1D**) and Patient-Derived-Xenograft (PDX) samples (**Figure 1E**) using FAK antibody. FAK is significantly higher in tumors than that in adjacent normal tissues. We also examined the correlation between FAK expression and OC patient survival using the Kaplan Meier Plotter database. High FAK expression is significantly correlated with patient poor overall survival (OS) and progression free survival (PFS) among 1435 OC patients including HGSC and endometroid (**Figure 1F**).

### FAK PROTAC Inhibits FAK Kinase Activity and Degrades Protein

**Figure 2:**
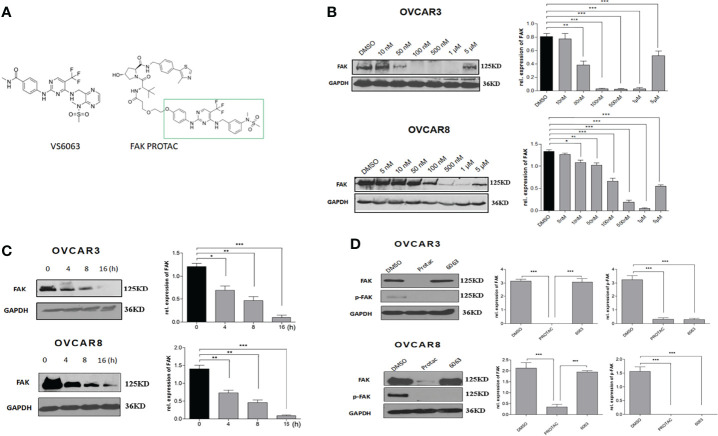
FAK PROTAC inhibits both FAK activity and degrades FAK protein. **(A)** FAK PROTAC binds FAK kinase inhibitor VS6063 and ubiquitin ligase E3. **(B)** WB analysis of FAK protein in both OVCAR3 and OVCAR8 cells following 24 h treatment at different doses. **(C)** WB analysis of FAK following 1 µM FAK PROTAC treatment at different time points. **(D)** WB analysis of FAK and pFAK in both OVCAR3 and OVCAR8 cells following 24 h treatment with 1 µM FAK PROTAC and VS6063. (*p<0.05; **p<0.01; ***p<0.001).

FAK PROTAC was designed and synthesized based on the parent FAK kinase inhibitor VS6063 (12). As shown in **Figure 2A**, FAK PROTAC links with VS6063 and a ubiquitin E3 ligase. VS6063 as a FAK kinase inhibitor targets FAK kinase activity and E3 ligase transfers ubiquitin to FAK, degrading FAK through polyubiquitination/proteasome pathway. To examine whether FAK PROTAC degrades FAK, we treated OC cells, OVCAR3 and OVCAR8, with different PROTAC doses for 24 h and performed WB to detect FAK protein levels. FAK was significantly degraded in OVCAR3 and OVCAR8 cells with a dose of 50 nM PROTAC and the most effective dose is 1 µM in both cell lines (**Figure 2B**). Next, we examined the time course of FAK degradation by treating both OVCAR3 and OVCAR8 cells with 1 µM PROTAC for 4, 8 and 16 h. FAK was significantly inhibited at 4 h and depleted at 16 h (**Figure 2C**). To compare the efficacy of FAK PROTAC and its parent FAK kinase inhibitor VS6063, we treated both OVCAR3 and OVCAR8 cells with 1 µM PROTAC or 1 µM VS6063 for 24h and then examined the phospho-FAK (pFAK) and total FAK by WB. As expected, VS6063 inhibits FAK activity but not total FAK protein, while FAK PROTAC inhibits FAK activity and depletes total FAK protein (**Figure 2D**).

### FAK PROTAC Is More Effective Than Its Parent FAK Kinase Inhibitor in Inhibiting OC Cell Growth, Migration, and Invasion

**Figure 3:**
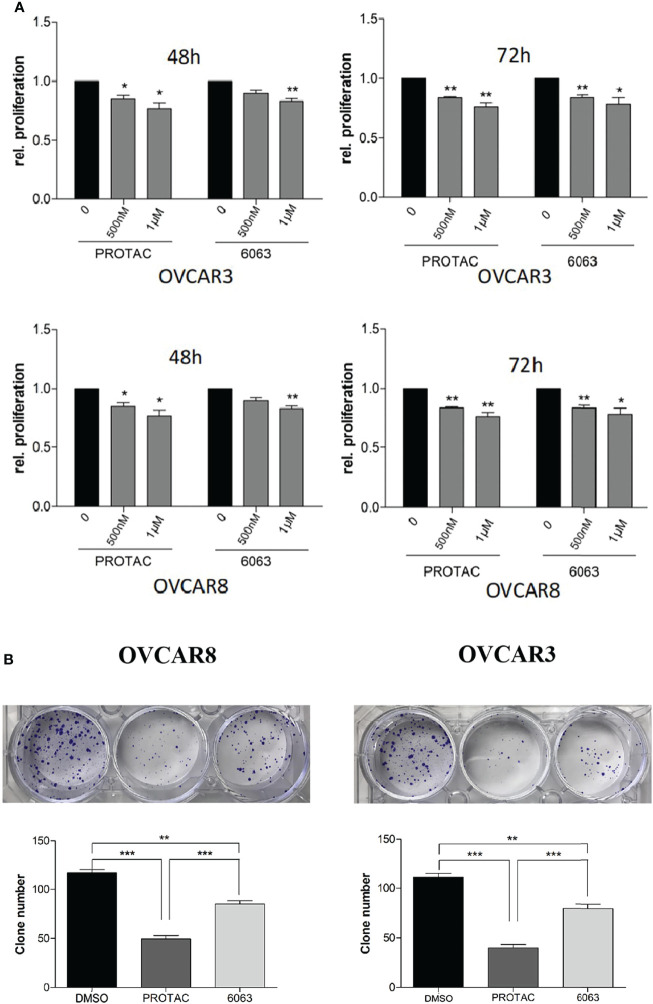
FAK PROTAC is more effective in inhibiting OC proliferation and clone formation. **(A)** Cell proliferation was detected by MTT assay following treatment of OVCAR3 and OVCAR8 with 1 µM FAK PROTAC and VS6063 for 48 or 72h. **(B)** Cell colonies were stained with crystal violet and counted following 0.5 µM VS6063 and 0.5 µM PROTAC treatment of OVCAR3 and OVCAR8 for two weeks. (*p<0.05; **p<0.01; ***p<0.001).

**Figure 4:**
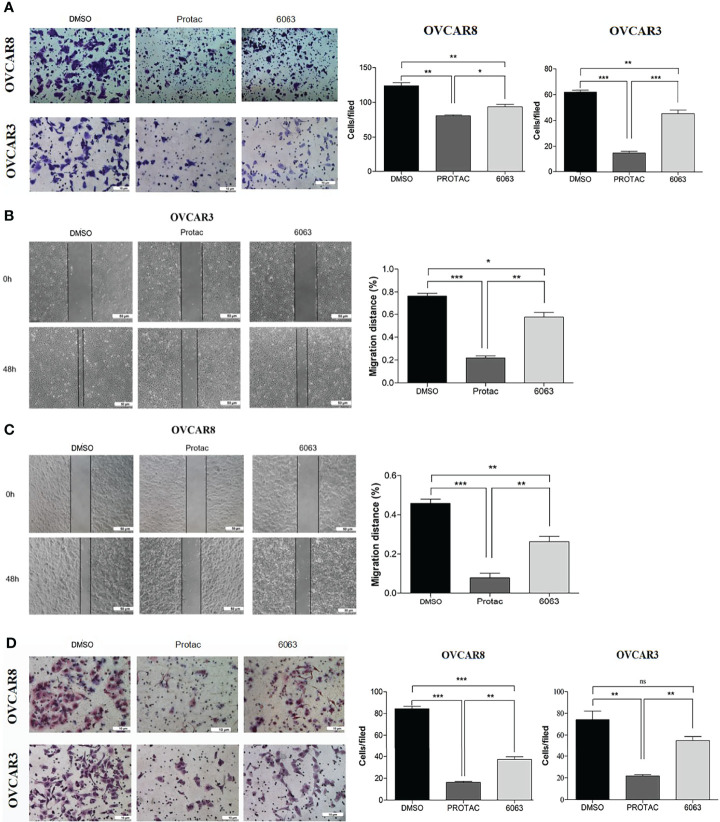
FAK PROTAC is more effective in inhibiting OC cell migration and invasion. **(A–C)** Cell migration in both OVCAR3 and OVCAR8 was examined using transwell plates **(A)** and wound healing assays **(B, C)**. Migrated cells in transwells were stained with crystal violet and counted from three different fields under microscopy. Migration in wound healing assay was calculated by comparing migrated distance at 48h to 0h. **(D)**. Invasion in OVCAR3 and OVCAR8 was examined using Matrigel coated transwell plates following 4h treatment with 1µM PROTAC or VS6063 and compared to vehicle treated cells. (*p<0.05; **p<0.01; ***p<0.001; ns, not significant).

To compare the functional differences of FAK PROTAC and FAK inhibitor VS6063, we treated OVCAR3 and OVCAR8 cells with FAK PROTAC, VS6063 or vehicle for 48 and 72 h and cell proliferation was determined by MTT assay. Both FAK PROTAC and VS6063 significantly inhibited cell proliferation and FAK PRTOAC is more effective than VS6063 at both time points (**Figure 3A**). To examine how PROTAC affects cell survival, we performed cell clonogenic assay after treating cells for two weeks with 1 µM PROTAC or VS6063. FAK PROTAC was more effective in inhibiting cell colony formation than VS6063 in both OVCAR3 and OVCAR8 cell lines (**Figure 3B**). We also assessed how FAK PROTAC affects cell migration and invasion by treating both OVCAR3 and OVCAR8 cells for 4 h with 1 µM FAK PROTAC and 1 µM VS6063. FAK PROTAC is more effective than VS6063 in inhibiting cell migration (**Figures 4A–C**) and invasion (**Figure 4D**) in both cell lines.

### FAK PROTAC Inhibits Kinase Activity and Disrupts the Interaction Between FAK and ASAP1

**Figure 5:**
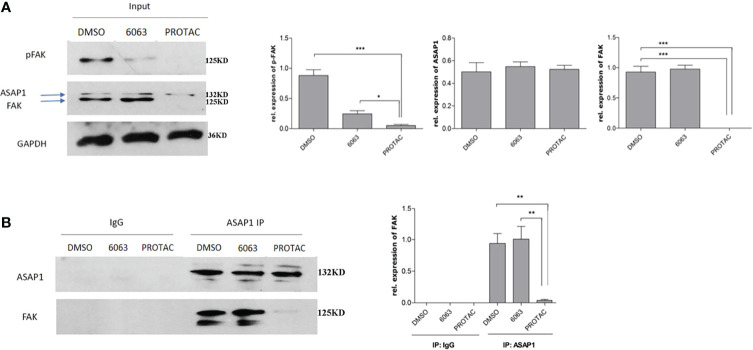
FAK PROTAC disrupts the interaction between FAK and ASAP1 in OC cells. **(A)** OVCAR3 cells were treated with 1 µM FAK PROTAC and VS6063 for 24 h, WB of input shows changes in pFAK, FAK, and ASAP1 in each group. **(B)** Protein complex was pulled down with ASAP1 antibody and then blotted with both FAK and ASAP1 antibody. (*p<0.05; **p<0.01; ***p<0.001).

We recently reported that FAK is co-expressed in OC and interacts with ASAP1 in OC cells (3). To understand whether FAK PROTAC blocks FAK kinase-dependent and kinase-independent pathways, we tested whether FAK PROTAC disrupts the interaction between FAK and ASAP1. We treated OVCAR3 cells with 1 µM PROTAC or 1 µM VS6063 for 24 h and then performed IP with ASAP1 antibody, followed by WB with ASAP1 and FAK antibodies. FAK protein was pulled down by ASAP1 in VS6063 treated cells but not in PROTAC-treated cells. Although both PROTAC and VS6063 inhibit pFAK (**Figure 5**), FAK PROTAC indeed disrupts the interaction between FAK and ASAP1. In contrast, VS6063 inhibits FAK activity as shown by reduced pFAK but does not disrupt the interaction between FAK and ASAP1.

### FAK PROTAC Inhibits OC Growth and Metastasis

**Figure 6:**
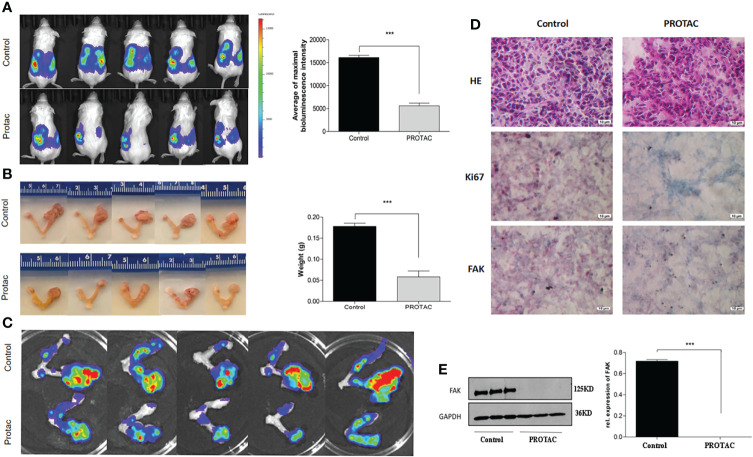
FAK PROTAC inhibits OC growth and metastasis. **(A, B)** Ovarian tumor growth and metastasis were shown by bioluminescence following three-week treatment with vehicle and FAK PROTAC in live animals **(A)** and ovarian tumors were also imaged **(B)** and weighted **(C)** in vehicle and FAK PRTOAC treated mice. **(D)** H.E and immunohistochemical staining against FAK and Ki67 of ovarian tumor sections in FAK PROTAC and vehicle treated mice. **(E)** WB analysis of FAK protein in ovarian tumors of FAK PROTAC and vehicle treated mice. (***p<0.001).

**Figure 7:**
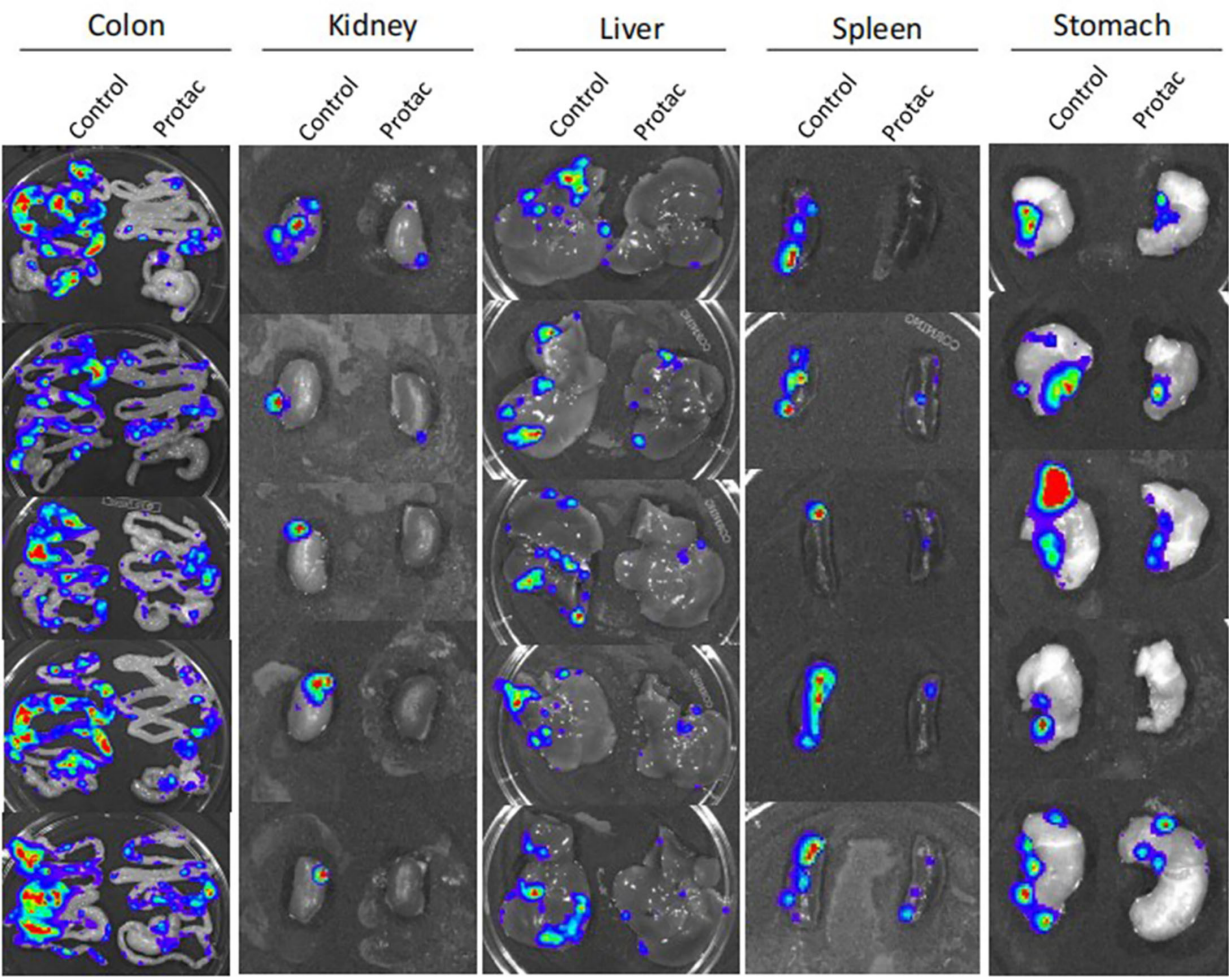
FAK PROTAC inhibits ovarian tumor metastasis. Metastatic tumors in colon, kidney, liver, spleen and stomach were visualized by bioluminescence imaging after three-weeks of FAK PROTAC and vehicle treatment.

We showed that FAK PROTAC is more effective than VS6063 in inhibiting cell growth, migration, and invasion. To test the efficacy of FAK PROTAC *in vivo*, we intrabursally injected OVCAR8 cells in immunocompromised mice and treated them with FAK PROTAC for three weeks. Ovarian tumor growth and metastasis was monitored weekly using Xenogen live animal imaging. FAK PROTAC significantly inhibited primary ovarian tumor growth as shown in bioluminescent images (**Figure 6A**). The decrease in dissected ovarian tumors by FAK PROTAC was also shown by tumor weight and bioluminescence (**Figures 6B, C**). To examine the FAK *in vivo*, we performed immunohistochemical staining. FAK was strongly stained in the cytoplasm of ovarian tumor sections of control mice but was weakly stained in ovarian tumor sections of mice treated with FAK PROTAC (**Figure 6D**). Ovarian tumors were also characterized by H&E staining (**Figure 6D**). The levels of FAK protein were diminished in ovarian tumors of mice treated with FAK PROTAC but was expressed in tumors of vehicle-treated mice with as shown by WB (**Figure 6E**). Tumor metastasis in different organs was also examined when mice were sacrificed, and FAK PROTAC significantly inhibits tumor metastasis as shown in intestine, liver, spleen, kidney, and stomach (**Figure 7**).

## Discussion

In this study we demonstrated that *PTK2* gene copy numbers are highly amplified and expression level is upregulated in multiple cancer types with the most upregulation in OC. High *PTK2* expression is well-correlated with poor OC patient survival. While FAK kinase inhibitors has not been successful in clinical trials, we showed that FAK PROTAC is more effective than its parent FAK kinase inhibitor VS6063 in inhibiting OC migration, invasion and metastasis. FAK PROTAC opens a new avenue in cancer therapy by degrading FAK, and thereby attenuating both FAK kinase-dependent and kinase-independent signaling pathways.

*PTK2* is located within the same genomic locus (8q24) as *ASAP1*, which is a region associated with aggressive cancer phenotypes, recurrence, and metastasis (28, 31, 32). The association of FAK with ASAP1 may play a significant role in tumor metastasis and chemoresistance. FAK was associated with ASAP1 in gastric cancer and associated with poor patient survival, indicating that FAK may play a synergistic role in gastric tumorigenesis and metastasis (25). ASAP1 was also associated with PD-L1 expression in pancreatic cancer fibrosis (33). The interaction between the SH3 domain of ASAP1 with the second prolin-rich domain of FAK has been found to contribute to focal adhesion assembly (23). We also reported that ASAP1 is highly expressed in OC and promotes OC invasion by facilitating EMT through activating EKR1/2 and AKT pathways (34). Although both *PTK2* and *ASAP1* were shown to be highly expressed in OC previously (35–37), it is yet unknown how the interaction between FAK and ASAP1 contributes to OC metastasis and metastasis. We recently reported that *PTK2* is co-expressed with *ASAP1* and FAK interacts with ASAP1 in OC based on HGSC dataset from TCGA database (3). Our finding indicates that the interaction between FAK and ASAP1 may also contribute to OC metastasis and chemoresistance. Therefore, it is important to target this interaction for effective OC therapy.

Due to ineffectiveness of FAK kinase inhibitors in cancer clinical trials, targeting FAK may require blocking both the kinase activity and kinase-independent function as a scaffold protein. FAK PROTAC was shown previously to be more effective than VS6063 in inhibiting breast cancer cell migration and invasion (12). FAK PRTOAC blocks FAK kinase activity and its scaffold function by degrading total FAK protein. Consistently, we found that FAK PROTAC is more effective than its parent VS6063 in inhibiting OC cell migration and invasion. In addition, we found that FAK PROTAC suppressed the proliferation of OC cells. Mechanistically, FAK PROTAC is a bifunctional molecule linked by FAK kinase inhibitor VS6063 and E3 ubiquitin ligase, which not only inhibit FAK kinase activity by reducing pFAK, but also disrupt the interaction between FAK and ASAP1 as shown by IP. FAK PROTAC degrades FAK, but not its interaction protein ASAP1 in OC cells. VS6063 only blocks FAK kinase activity without disrupting the interaction between FAK and ASAP1. Our data indicate that FAK PROTAC is indeed more effective than its parent inhibitor VS6063 by also disrupting FAK kinase-independent scaffold activity. Interestingly, we observed the “hook effect” that FAK was slightly rebound due to forming a binary, not the ternary complex required for effective protein degradation at 5 µM high dose in OC cells. The similar phenomenon was observed in human prostate tumor cells (12, 38).We also tested the efficacy of FAK PROTAC using orthotopic OC mouse models and showed marked activity *in vivo* to inhibit ovarian tumor growth and metastasis, thus potentially transform the clinical ovarian cancer therapy. However, although PROTAC showed promising in cancer clinical trials, it is important to further investigate the off-target effects, stability, and toxicity *in vivo*. We will further test its efficacy in treating chemotherapy drug resistant cancers through combination therapy.

In summary, we present experimental evidence that FAK PROTAC is more effective than its parent FAK inhibitor by blocking both FAK kinase activity and its scaffold protein function through disrupting the interaction between FAK and ASAP1 in OC.

## Data Availability Statement

The original contributions presented in the study are included in the article/**Supplementary Material**. Further inquiries can be directed to the corresponding authors.

## Ethics Statement

The studies involving human participants were reviewed and approved by UTHSC IRB. Written informed consent for participation was not required for this study in accordance with the national legislation and the institutional requirements.

## Author Contributions

Conceptualization/designs: JY, WZ, and ZC; Data collection and analysis: XH, WZ, GZ, and RN; JY, WZ, HW, PD, TT, LP, and ZC: wrote and edited the manuscript. All authors contributed to the article and approved the submitted version.

## Funding

This study was partially supported by 1R21CA216585-01A1 (JY) from the NCI; a CORNET award from UTHSC (JY and WZ); and a grant form the National Natural Science Foundation of China (81902332, XH).

## Author Disclaimer

The content is solely the responsibility of the authors and does not necessarily represent the official views of the NIH.

## Conflict of Interest

The authors declare that the research was conducted in the absence of any commercial or financial relationships that could be construed as a potential conflict of interest.

## Publisher’s Note

All claims expressed in this article are solely those of the authors and do not necessarily represent those of their affiliated organizations, or those of the publisher, the editors and the reviewers. Any product that may be evaluated in this article, or claim that may be made by its manufacturer, is not guaranteed or endorsed by the publisher.

## Abbreviations

OC, ovarian cancer; FAK, focal adhesion kinase; PTK2, protein tyrosine kinase; PROTAC, Proteolysis Targeting Chimeric Molecule; POI, protein of interest; FAT, focal adhesion targeting; SH, proline-rich; FERM, 4.1-ezrin-radixin-moesin; NES, nuclear export signal; ASAP1, ADP ribosylation factor ARF GTPase-activating protein GAP containing SH3, ANK repeats, and PH domain; PH: pleckstrin homology; NSG, NOD scid gamma; WB, western blot; HGSC, high-grade serous carcinoma; PDX, Patient-Derived-Xenograft.
