# Supplementary material for: FAK PROTAC Inhibits Ovarian Tumor Growth and Metastasis by Disrupting Kinase Dependent and Independent Pathways
Source: Front Oncol. 2022 Apr 28;12:851065. doi: 10.3389/fonc.2022.851065 (PMC9095959; doi:10.3389/fonc.2022.851065)
Supplement: Supplementary file 1 [file DataSheet_1.docx]

Supplementary Material

Supplementary Figure


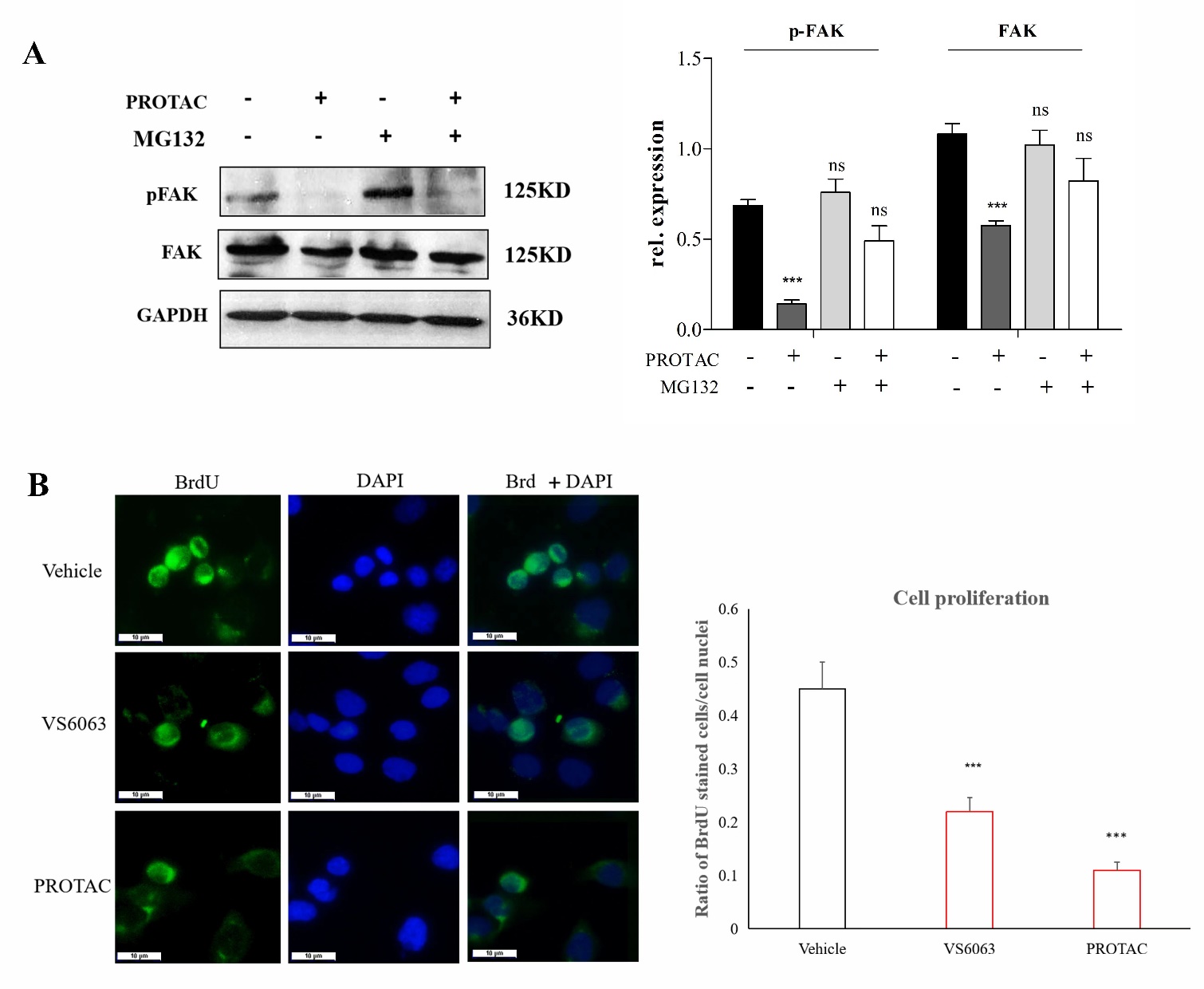


Supplementary figure 1. Changes in Phosphorylated FAK and total FAK proteins after treatment of PROTAC and MG132. OVCAR3 cells were treated with MG132 (5 µM) overnight and then with 1µM PROTAC for additional 3 h. Western blot was used to determine the protein expression of pFAK and FAK. Right panel shows the quantification of the blots. (****p*<0.001 compared to untreated group).


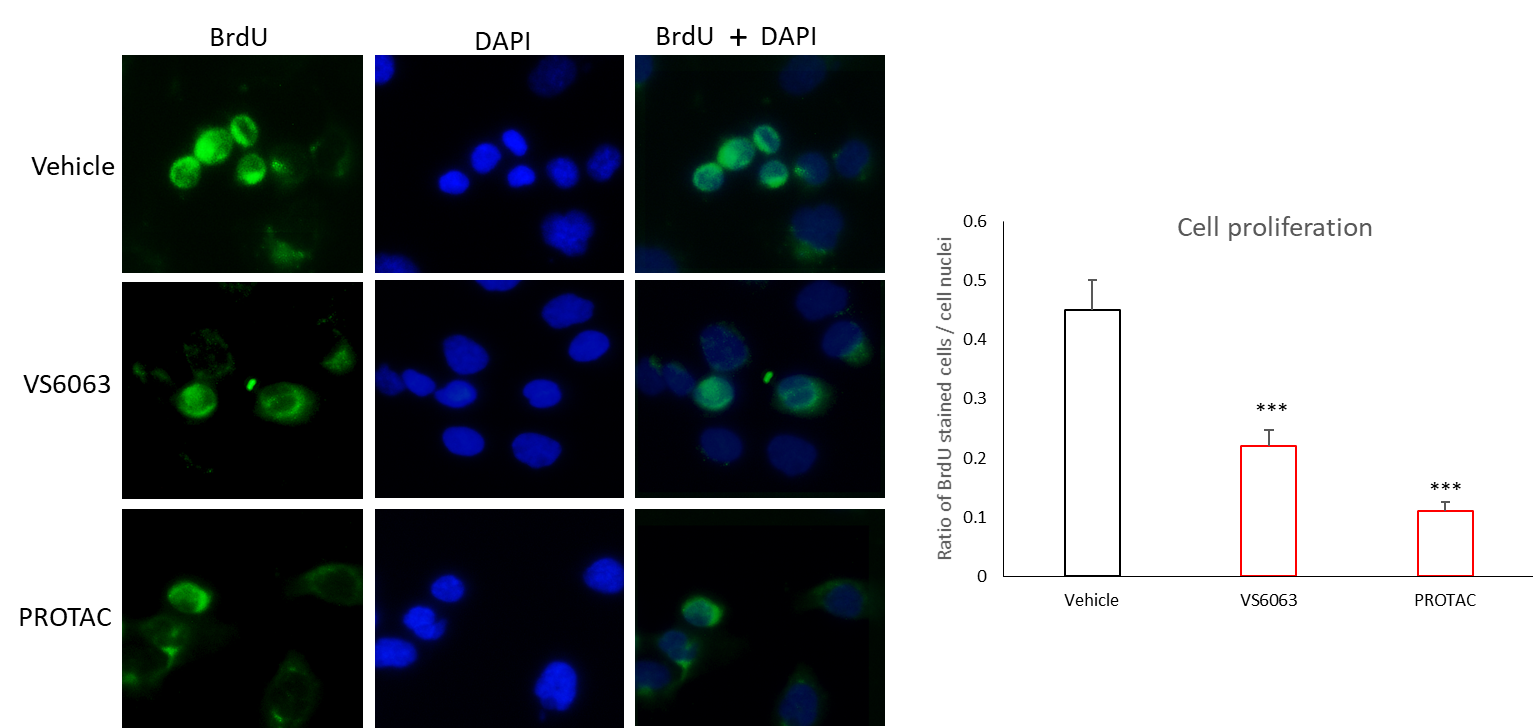


Supplementary figure 2. BrdU proliferation assay of OC cells treated with VS6063 or PROTAC. OVCAR3 cells were labelled with BrdU for 12 h and then detected by BrdU antibody. Right panel shows the quantification of BrdU+ cells. DAPI was used to label nuclei. (****p*<0.001 compared to vehicle treated group).
